# Software review: The JATSdecoder package—extract metadata, abstract and sectioned text from NISO-JATS coded XML documents; Insights to PubMed central’s open access database

**DOI:** 10.1007/s11192-021-04162-z

**Published:** 2021-10-24

**Authors:** Ingmar Böschen

**Affiliations:** Psychological Methods and Statistics, Institute of Psychology, University Hamburg, Von-Melle-Park 5, 20146 Hamburg, Germany

**Keywords:** Meta-research, Text extraction, Text mining, PubMed central, Software

## Abstract

*JATSdecoder* is a general toolbox which facilitates text extraction and analytical tasks on NISO-JATS coded XML documents. Its function *JATSdecoder()* outputs metadata, the abstract, the sectioned text and reference list as easy selectable elements. One of the biggest repositories for open access full texts covering biology and the medical and health sciences is PubMed Central (PMC), with more than 3.2 million files. This report provides an overview of the PMC document collection processed with *JATSdecoder()*. The development of extracted tags is displayed for the full corpus over time and in greater detail for some meta tags. Possibilities and limitations for text miners working with scientific literature are outlined. The NISO-JATS-tags are used quite consistently nowadays and allow a reliable extraction of metadata and text elements. International collaborations are more present than ever. There are obvious errors in the date stamps of some documents. Only about half of all articles from 2020 contain at least one author listed with an author identification code. Since many authors share the same name, the identification of person-related content is problematic, especially for authors with Asian names. *JATSdecoder()* reliably extracts key metadata and text elements from NISO-JATS coded XML files. When combined with the rich, publicly available content within PMCs database, new monitoring and text mining approaches can be carried out easily. Any selection of article subsets should be carefully performed with in- and exclusion criteria on several NISO-JATS tags, as both the subject and keyword tags are used quite inconsistently.

## Introduction

Scientists from all over the world cumulate knowledge to describe and better understand the causes and effects in a very wide range of research areas. Nowadays, hundreds of thousands of research-related documents are published every year in an incredibly wide range of disciplines, subjects and journals. Scientific findings are mostly published in peer-reviewed journals that are more and more willing to share their articles, or at least parts, without charging readers a fee in so-called open access journals. Combined with the increasing computational power of personal computers it is possible for anyone to purchase and deal with essentially big amounts of textual data as source of information or research interest.

The Science-Metrix report (Science-Metrix Inc. 2018) quantifies the availability of scientific research findings in the form of open access versions to be about one half of all results published. About one paper out of four is freely made available by the publishers themselves (gold open access), most of the time on their own websites but also frequently mediated by websites such as PubMedCentral, SciELO in some Romance-language countries, and JStage in Japan (Science-Metrix Inc. 2018).

Aside from the benefits of many scientific findings and information being made publicly available, selection and identification problems arise. The identification of articles topic relatedness is a key part for any researcher running a literature search. In order to make an informed judgement about a specific research topic and meta-analytical questions, the identification and preselection of relevant studies as well as the extraction of relevant information are more and more time-consuming tasks as the text corpus is constantly growing. Search engines can help to identify studies by metadata like title, author and/or keywords, but rarely by search tasks on the full textual content nor by automatically extracted methodological study features.

### Knowledge discovery with text mining

In a nutshell, text mining (TM) is the process of discovering and capturing knowledge or useful patterns from a large number of unstructured textual data (Zheng and Benoit 2019). It is an interdisciplinary field that draws on data mining, machine learning, natural language processing, statistics, and more. (Zheng and Benoit 2019).

Current research in the area of text mining tackles problems of text representation, classification, clustering, information extraction or the search for and modelling of hidden patterns (Hotho et al. 2005). A wide variety of biomedical text-mining tasks are currently being pursued, such as entity recognition (e.g. finding mentions of genes, proteins, diseases) and extraction of molecular relationships (e.g. protein-protein interactions) (Gerner et al. 2010).

The increasing calculation power of computers and database capabilities combined with the focused development of neural networks and machine learning algorithms make some contemporary systems that process natural language *’artificially intelligent’*. These systems can discover hidden patterns and relationships in huge amounts of text that a person could never read or summarize during their lifetime.

For most computational text analysis, full texts must be tokenized into smaller, more specific text features, such as words or word combinations (Welbers et al. 2017). Text preparation often involves sentence and word annotations, uniformization and dimension reduction like stemming or lemmatization. Further, text may be lowerized (converting capital to lower letters) and cleaned up by removing arbitrary letters and/or words, extra spaces, numbers and/or punctuations.

Recent years have seen rapid development in algorithm based text clustering, extraction and contextual analysis methods. To name just a few applied projects that recently made use of text mining methods on scientific content:

Watanabe (2021) developed a semi-supervised text analysis technique for discovering new research domains. Westergaard et al. (2018) showed that a contextual text analysis on full text sources is substantially outperforming and more informative than an analysis on abstracts only. Anderlucci et al. (2017) described the evolution of recent scientific research topics in statistics from 1970–2015 using a semi-supervised mixture model. Nuijten et al. (2016) developed the program *statcheck* to automatically detect potential errors in documents that report statistical result in APA style. Head et al. (2015) extracted reported *p*-values out of the PMC article collection and found substantial support for the presence of p-hacking. (Gerner et al. 2010) developed LINNAEUS, a species name identification system for biomedical literature.

This report draws a quantitative bibliometric profile of the PMC article collection by extracting metadata and text parts with *JATSdecoder*. The unified structure and letter representations may be useful for any text mining project on NISO-JATS coded content.

### PubMed central and NISO-JATS

The PubMed Central$\circledR$ (PMC) is a free full-text archive of biomedical and life sciences journal literature at the U.S. National Institutes of Health’s National Library of Medicine (NIH/NLM) (PubMed-Central 2020) that does not act as a publisher itself. It allows full online access to more than three million documents that consist of abstracts, full text research papers and materials. PMC provides systematic download opportunities to access its full content or only subsets (the PMC OAI service and the PMC FTP service) that have a Creative Commons BY or similar license. The full bulk download service was disabled on 18th of March 2019 and the articles are now offered as Commercial- and Non-Commercial-Use bulk packages. All documents are provided as eXtensible Markup Language (NXML) or JavaScript Object Notation (JSON) files.

PMC’s NXML files follow a well documented structure called NISO-JATS (National Center for Biotechnology Information 2014). It makes most article content and meta information easily accessible if used adequately by the editorial and/or technical implementation team. The NISO-JATS tag set is the most effective and widely used archival format for journal articles (PubMed-Central 2020). NISO-JATS is a flexible HTML coding system that defines 277 elements and their use. Though Comeau et al. (2018) point out that NISO-JATS is not designed to aid text mining, as the text appears at different levels and in various structures, the great benefit of its structure is demonstrated here by successfully extracting core article metadata and content with *JATSdecoder*.

The Content ExtRactor and MINEr *CERMINE* (Tkaczyk et al. 2015) is a sophisticated PDF to text conversion tool that extracts metadata, full text and parsed references from scientific articles and outputs a NISO-JATS coded XML file that is processable with *JATSdecoder*. *JATSdecoder* contains some correction procedures to deal with *CERMINE* specific and general PDF compilation problems (e.g. coding of special characters and operators).

### JATSdecoder

The R package *JATSdecoder* (Böschen 2021) is an infrastructure software that contains a set of functions to extract and unify content from scientific literature coded in NISO-JATS format. Its function *JATSdecoder()* bundles all supplied functions to extract metadata, sectioned text and reference lists. Its function *study.character()* performs multiple text extraction tasks on specific methodological characteristics like statistical methods applied, $\alpha$-error, correction methods and all reported statistical test results within the text. The p-values of extracted test statistics are recomputed to facilitate a manual check on consistency and reporting style (Böschen 2021b).

*JATSdecoder* makes use of regular expressions in simple search and text manipulation functions like: *grep()*, *gsub()*, *substr()*, *strsplit()*, *paste()*, *tolower()* to extract the content of the targeted tag. In standard mode, *JATSdecoder*’s function *letter.convert()* transforms and unifies all hexadecimal and many HTML letter representations to Unicode letters. Distinct hyphens, spaces, operators and many other special characters are unified to a character that is part of any Western computer keyboard to facilitate further text processing tasks. For example, more than 20 codings for distinct spaces are unified to a standard space. Spacing errors are corrected, hyperlinks and HTML formatting tags are removed. In standard mode, *JATSdecoder()* unifies name and country representations to facilitate post-processing and search tasks. Therefore, the *JATSdecoder* output is not an exact representation of the original content but easy to post-process and analyze.

The NISO-JATS tags <title>, <journal>, <type>, <abstract> are simple uniformly used metadata tags. Their content can be extracted easily with simple regular expressions. Tags that are less structured and more flexible to use demand more sophisticated processes. The use of the <subject> and <kwd> (keyword) tags differs in terms of their technical implementation (with/without sub-subjects) and summarizing precision. Within the <history> tag, contributors are comparatively free to choose the precision and coding of each files publication history. *JATSdecoder*’s function *get.history()* extracts the supplied date stamps and calculates the first publishing date and year of publication if possible. Its function *get.text()* exports the contained text sectionwise. Since text sectioning is implemented inconsistently, sections and subsections are not differentiated. The sectioned text can be returned as floating text or vector with sentences for every section. *get.text()*’s argument *’sectionsplit’* can be used to split the text into main sections by defining an individual pattern vector. Section names that contain the defined pattern/s are combined with all following sections until the next matching pattern appears within the section names.

Listing 1 displays an example *JATSdecoder()* output for the article by Blanca et al. (2018). Here the text is split into four sections by setting the argument ’sectionsplit’ to *’c(’intro’, ’method’, ’result’, ’discussion’)’*. *JATSdecoder*’s function *text2sentences()* is used to return the abstract as sentence vector, the main text is returned as floating text per section split.

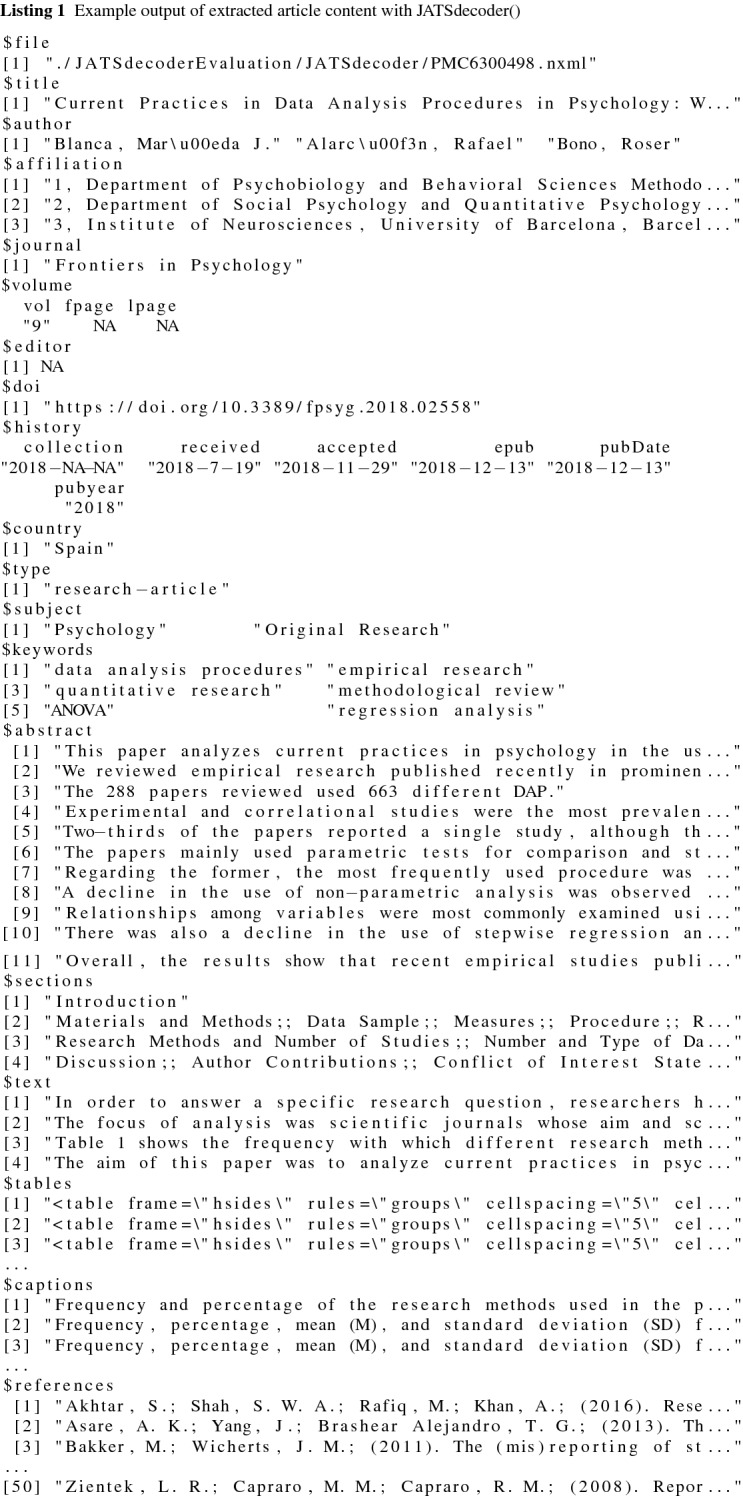



## Method

PubMedCentral’s ftp server (ftp://ftp.ncbi.nlm.nih.gov/pub/pmc/) was used on 01.01.2021 to bulk download all available documents in NXML format. After unzipping all folders *JATSdecoder()* is used to extract the article elements title, journal, history, author, editor, type, subject, keywords, affiliation, country, section names, full text and reference list.

The relative frequency of successfully extracted content is reported for every tag and different time intervals to display the development of the availability and consistency in use of the extracted tags over time. Each meta tag is analyzed globally to further explore the full corpus and demonstrate new monitoring techniques enabled with *JATSdecoder*. Descriptive analyses of low dimensional tags are performed with frequency tables and bar plots. Content of higher dimensional tags (subject, keywords, author and editor names) is reduced to the most frequent labels and presented in word clouds (Fellows 2018). As the representation of information in text data is mostly unstructured, some inconsistencies and general burdens for search tasks and text analytical approaches on PMC’s database are outlined.

An AMD@Epyc 7452 32-core processor with 256GB ram memory running with Linux Ubuntu 20.04.1 LTS is used. All tag extractions and analytical computations are performed with the open source statistic software R R Core Team (2020) and its package *JATSdecoder* (Böschen 2021). Multi-core processes are applied with the R package *future* (Bengtsson 2020) to speed up the extraction.

The syntax and extracted data to reproduce or update this analysis can be accessed at: https://github.com/ingmarboeschen/JATSdecoderEvaluation/.

## Results

### Summary and NISO-JATS tag use over time

As of January 1st, 2021, the PMC database contained 3,236,331 articles published by 16,789 journals with a total file size of 260.17 GB. Table 1 displays the overall frequency of successfully extracted tags and for some subsets of publishing periods. Tags that should contain text (e.g.: title, abstract, author) are treated as not present if the tag is detected but does not contain any content. Abstracts and text parts with less than 30 characters are treated as not present.

**Table 1:** Relative frequency of extracted tag use over time

Content	Total use	[1781; 2000]	(2000; 2005]	(2005; 2010]	(2010; 2015]	(2015; 2020]
Abstract	87.9%	41.3%	71.7%	87.9%	88.9%	92.2%
Affiliation	94.8%	38.4%	89.2%	96.4%	98%	98.4%
Author	96.9%	56.2%	96.9%	98.9%	99.2%	99.2%
Country	82.9%	15.4%	74.7%	86.9%	88.4%	86.2%
Doi	100%	100%	100%	100%	100%	100%
Editor	12.6%	0.1%	1.7%	10.2%	18.2%	11.5%
History	100%	100%	100%	100%	100%	100%
Journal	100%	100%	100%	100%	100%	100%
Keywords	59.9%	8%	42.2%	35.1%	52.9%	72%
References	90.3%	9.7%	69.6%	92.2%	94.6%	96%
Sections	88.6%	8.9%	71.9%	91.3%	93.6%	93.6%
Subject	99.8%	100%	99.9%	99.7%	99.8%	99.8%
Text	88.5%	8.8%	71.9%	91.2%	93.5%	93.5%
Title	100%	99.9%	99.8%	100%	100%	100%
Type	100%	100%	100%	100%	100%	100%
Volume	48.7%	93.4%	82.4%	77.1%	54.9%	36.9%
Total *n*	3,235,065	171,394	52,488	239,952	924,154	1,846,979

The title, journal, history, type and subject tag contain extractable content in nearly all the documents. Material that has been published since the year 2000 tends to have much higher amounts of extractable tags in general. Within content that was published before 2000 the affiliation and country tags are rarely used, whereas nowadays, they are present and consistently used in the majority of content. Keywords are only present in about half of the articles. In 2018 about two thirds of all articles supplied keywords additionally to their subject tag. The editor tag is rarely used and shows no trend towards omnipresence in recent releases either. With 508,834 cited documents, 2020 is the most frequent year of release.

### Document type

**Fig. 1:**
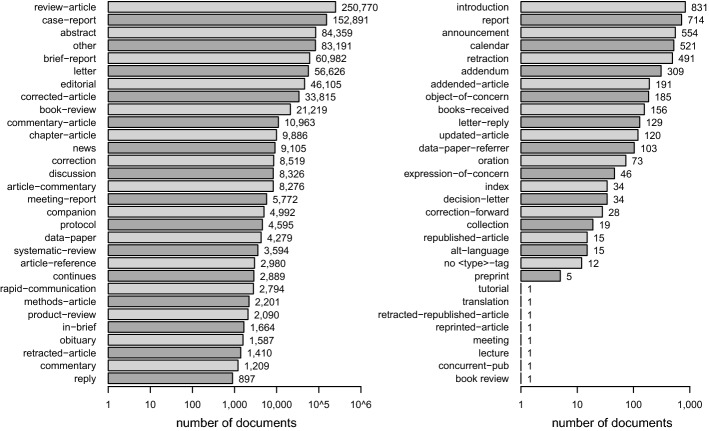
Absolute frequency distribution of article types

The <type> tag is consistently used to specify the global article content with one categorizing label only. 12 articles do not contribute a <type> tag. Figure 1 displays the frequency distribution of the 60 discovered <type> tag specifications on a log transformed x scale.

The vast majority of publications is labeled as ’research article’ (72%, *N* = 2,343,752), followed by reviews (8%, *N* =250,770) and case-reports (5%, *N* = 152,891). There are 42,362 articles declared as corrections (’corrected-article’, ’correction’, ’correction-forward’) and 1902 articles tagged as retraction (’retracted-article’, ’retraction’). Some type categories overlap. An inspection of the title tag reveals that 20,694 documents tagged as ’research-article’ have a title containing the search terms ’systematic review’ or ’literature review’. Further, 3049 documents type tagged as ’research-article’ contain the search term ’review’ inside the subject and 3987 inside the keyword tag, with 258 articles containing ’review’ in both tags.

### Involved journals

The <journal> tag is used in all non-empty files and is easy to extract. Table 2 shows the absolute and relative frequency of the number of provided articles per journal. Out of 16,789 journals, 61.1% provide only 10 or fewer documents. There are 33 journals (0.2%) that provide more than 10,000 documents each. Taken together, these 33 journals contribute 971,674 articles (30%) to PMC.

**Table 2:** Absolute (h) and relative (f) frequency of journals supplying n articles

*n*	1	2–10	11–100	101–1001	1001–$10^4$	10,001–$10^5$	$>10^5$	Sum
*h*(*n*)	5,078	5,178	3,553	2,461	486	31	2	16,789
*f*(*n*)	.302	.308	.212	.147	.029	.002	.000	1

**Fig. 2:**
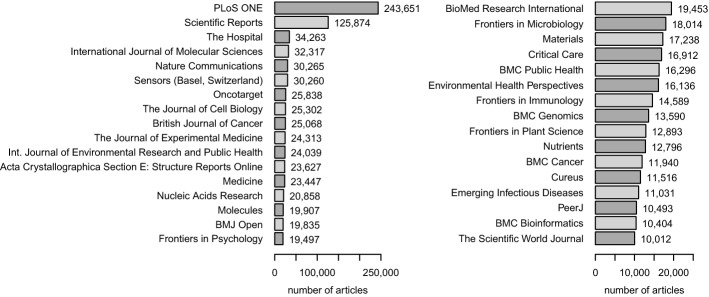
Total number of published articles for journals with >10,000 articles

Figure 2 displays the absolute number of documents by those journals that are identified more than 10,000 times. *PLoS ONE* is the most prominent publisher, followed by *Scientific Reports* and *The Hospital*.

### Publication history

The publication history is usually stored within a <date> tag inside the <history> tag. A pre analysis revealed that 14 different time stamp labels are being used. *JATSdecoder()* extracts the earliest publishing date (’pubDate’) and year (’pubyear’) out of six possible publication date stamps to facilitate selection procedures.

Publishers support different and inconsistently precise information about the article’s publication history. Only 1266 documents do not contain extractable date stamps. Table 3 displays the frequency of use and captured date range for each of the identified date types and standardized publishing dates, created by *JATSdecoder()*. Missing <month> and <day> tags within a history date stamp were set to 1.

**Table 3:** Frequencies of use and time spans of extracted history date stamps

Date type	Relative use (%)	Earliest date	Latest date
Accepted	77.9	1882-3-15	2100-1-14
Collection	62.7	1973-10-NA	2021-NA-NA
Ecorrected	0.1	2010-10-04	2020-9-8
Epreprint	0.6	2005-10-10	2020-NA-NA
Epub	85.5	1957-10-01	2021-1-1
Nihms_submitted	0.9	2006-2-23	2020-9-9
pmc-release	27.5	1799-11-NA	2021-9-21
ppub	36.1	1781-1-NA	2022-NA-NA
Print	0.7	1989-NA-NA	2020-NA-NA
pub	1.1	2009-4-01	2020-NA-NA
Received	78.7	1856-1-19	2097-10-22
Retracted	0	2018-6-01	2018-8-27
Rev-recd	18.6	1800-1-16	2088-10-30
Submitted	0	2019-10-29	2020-9-7
PubDate$^\star$	90.4	1802-08-01	2021-11-01
Pubyear$^\star$	100	1781	2021

*Computed by *JATSdecoder()*

The oldest publications listed are 140 books and articles published in 1781, which are distributed by the *The London Medical Journal* as scanned PDF documents.

Since then, documents from every year are available. About 82.2% of all articles were published after January 1st 2000, when the boom of the open access movement started. The ’retracted’ and ’submitted’ date stamps are used very rarely.

Table 3 indicates that there are obvious errors in date stamps of some documents, as they contain future dates. Further, the calculated publication date, the accept- and receive date can serve for another analysis of errors in date stamps. Calculating the time to accept (accept date–receive date) and time to publish (publishing date–accept date) reveals that there are 2,310 documents with an accept date prior to their receive date and 12,303 articles with a publishing date prior to their accept date.

### Contributor

The <contrib> tag is used to store one or multiple <surname> and <name> tags of author and or editor name/s, as well as involved affiliations and contact addresses.

The representation of given names differs across journals. Some journals provide given and surnames in fully capital letters, others use one letter abbreviations of all or only given middle names. Some names with an Asian origin are provided with Latin letters, others with Asian characters. *JATSdecoder*’s functions that extract the author/s and editor/s name convert fully capital letter names to a representation with an initial capital letter only and enable shortening of given names to a one letter abbreviation if desired.

#### Authors

It is widely known that a considerable proportion of authors share the same last name and first initial (Falagas 2006) which is a big problem for correctly identifying individuals and networks in PMCs database. Besides that, the use of the Western-driven (surname/family name|given/first name|middle initial) system is particularly problematic for Asian biomedical researchers in general: Japan, China and especially Korea, where only a few surnames predominate and middle names often do not exist (Harrison and Harrison 2016).

**Fig. 3:**
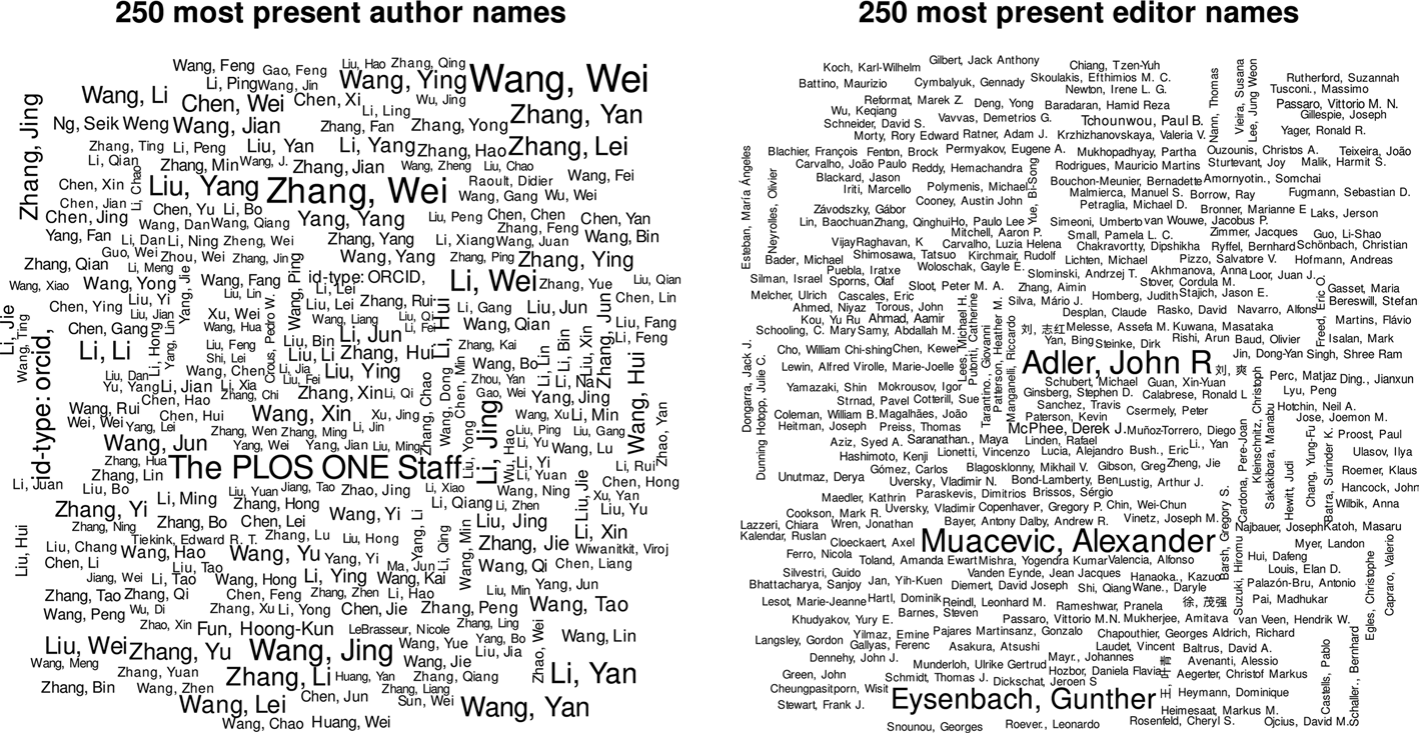
Wordclouds of 250 most present author and editor names

This phenomenon becomes obvious in Fig. 3 which displays the 250 most frequent author names in PMC. It is obvious that most of the names have an Asian origin. First and last names are pretty short and the same names occur quite often, resulting in a lot of name siblings. All displayed author names appear at least 342 times with a maximum of 3,643 detections of ’Wang, Wei’, the most present author name in PMC documents.

Several registers that supply a nomenclature for a clear author distinction exist. Harrison and Harrison (2016) have highly recommended their use. A comparably small but steadily increasing amount of articles in PMC supplies an author identification number for at least one author. 9.8% of all articles contain at least one ID coded author name, and out of these 20% have ID tagged at least half of the contributing authors. The *Open Researcher and Contributor ID* (ORCID) is the far most commonly identified identifier with 696,416 distinct IDs. 186 authors are listed with n *sciprofiles* ID, 53 with a *zoobank* ID, 10 authors are coded with their *twitter* names. In 2020 43.8% of all documents had at least one author ID tagged. Before 2009 no document contained any ID tagged author.

For a long period of time, it was quite usual to publish in very small groups or alone. But the amount and intensity of author collaborations has been growing steadily for decades. In 2020 the median of author group size grew up to 5 persons, meaning that more than 50% of content is published by researcher groups equal to or larger than 5. There are 547 articles with more than 500 authors. A manual inspection of the titles and types of these articles reveals that there are mostly two categories of content with such big author groups. 282 (51.55%) of these articles are published by international collaborations covering topics like ’atlas’, ’detector’, ’proton’, ’collision’, ’jet’ and/or ’particle’. The remaining documents with such large author groups are mostly abstracts and poster collections from congresses or meetings.

#### Editors

The editor information is the least often identified meta tag. 407,359 (12.59%) of all documents contain an extractable editor name. Only 19 of all 42,455 unique editor names are represented by an author identification number. Compared to the contributing author names, most editor names have a Western-driven origin and mostly refer to the same person. The problem of the indistinguishability of editor names will become worse as soon as more journals with Asian named editors provide content to PMC.

91.9% of all documents that supply editor information were edited by one person, 6.3% list two editors. One comparably large group of involved editors was identified in the German book about infectious diseases ’Infektionserkrankungen’ (Bialek et al. 2007) with 121 listed editors.

#### Affiliation

The <aff> tag is usually stored within the <contrib> tag and used to supply information about the affiliations of contributing author/s. The data is stored with differing precision and technical implementation. Some documents supply the full name of a department within an institution, an abbreviation as well as full contact details, while in others, the main institution name is supplied with a country location. Some distributors use tags to locate special elements of affiliation details (name, address line, country) while others supply this data in an untagged line. Further, the naming of one and the same institution is not completely coherent (e.g.: ’University of Oxford’ and ’Oxford University’). Therefore, a global analysis of the affiliation tag is not reported here.

Since the storing of detailed affiliation data differs widely, *JATSdecoder()* removes all HTML-tags within the <aff> tag and outputs a string with comma separated specifications. Any analysis of the affiliation tag should involve a pre-targeting of specific institutions and further post-processes like uniformization of name representations and removing redundant content like postal codes.

### Subjects and keywords

The <subject> tag is used within an <article-category> tag or stands alone to describe the document content or type. Without any uniformization 66,118 distinct subject labels are identified. About two-thirds of all documents contain one classifying element, which is mostly similar to the <type> tag (e.g.: type: ’research-article’, subject: ’Research Article’) but partially further specifies the document’s type. Some contributors make intense use of the subject specification. 0.5% of documents contain more than 38 subjects, with a maximum of 739 declared subjects’.

**Fig. 4:**
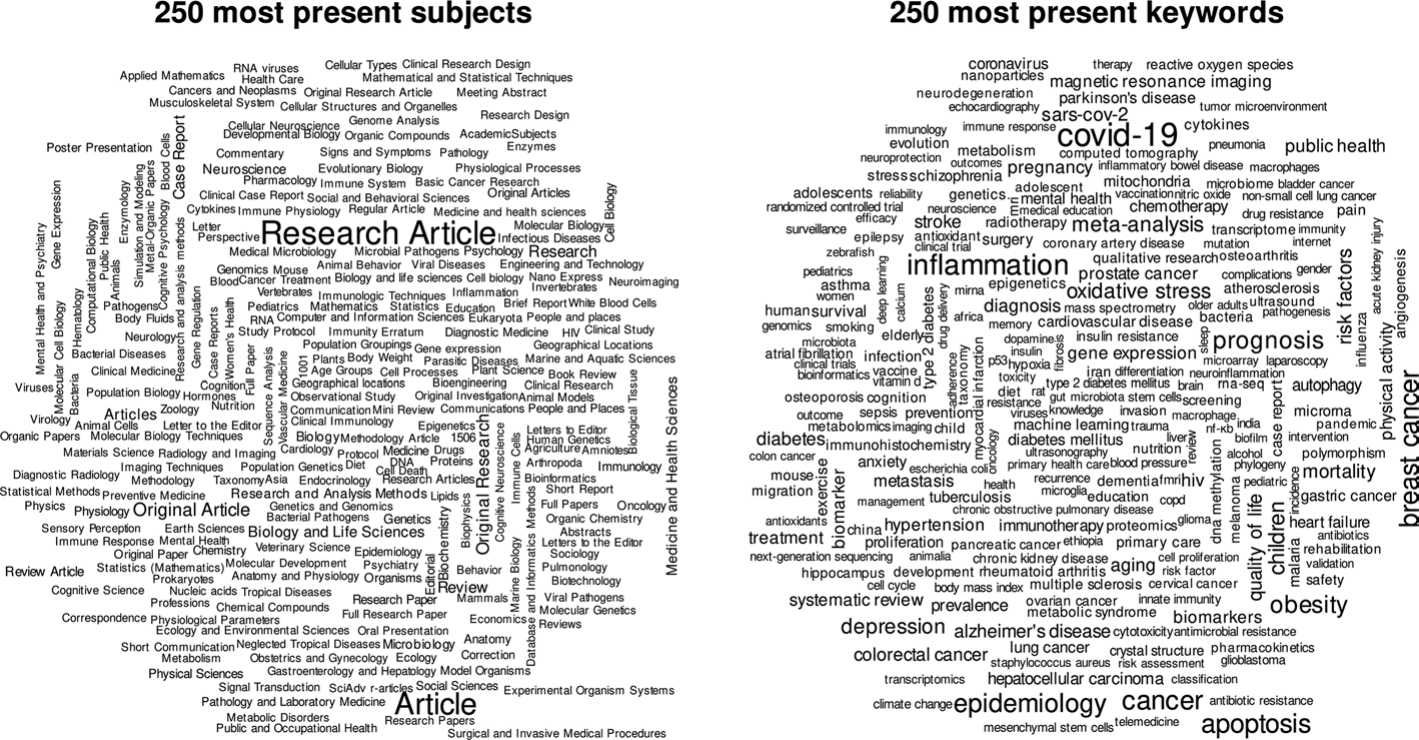
Wordclouds of 250 most frequent <subject> and <kwd> tags

The <kwd> tag is explicitly designed to contain keywords that briefly describe the article’s content. Within the last two decades, keywords have developed from a rarely to very often used specifier of documents. As Table 1 indicates, keywords are only used in about half of all documents. In 2020 the relative frequency of keyword-tagged-documents peaks at 78.4%. An inspection of overly intense keyword uses reveals that some documents contain the abstract’s text inside the <kwd> tag. Figure 4 displays the 250 most frequently extracted subject and keyword labels in separate word clouds. Bigger words indicate a higher frequency of use.

The most urgent and influential research topic in 2020 surely is the Covid-19/SARS-Cov-2 pandemia (*N* = 31,549). Within only one year, it has become the most common keyword specified within all PMC documents.

### Country of origin

The <country> tag is used to store the involved author/s and/or affiliation/s country of origin. If no tag is used to store this information, *JATSdecoder()* performs a dictionary search at the end of each extracted affiliation address and returns a vector with uniquely identified country names. Some country namings that are different to the standard country name (e.g.: Peoples Rep. of China, UNITED STATES, UK) are unified by a manually generated list of pre-identified country namings to facilitate any post-processing on world maps. Overall, 208 out of 243 countries of origin have been detected. 82.9% of all documents contain at least one extractable country of origin. 30.8% of these documents have been released by international author groups, as they contain more than one extractable country of origin.

**Fig. 5:**
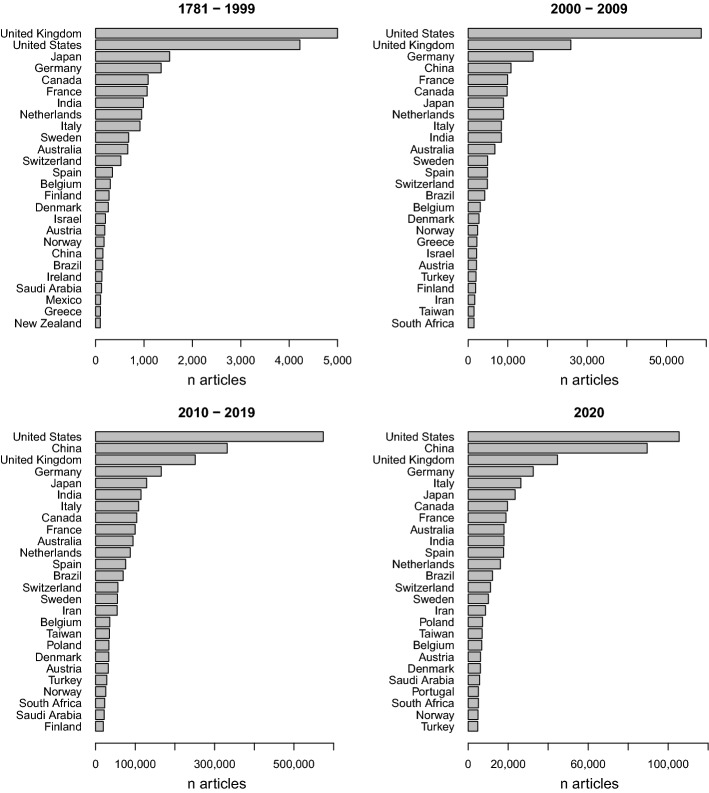
Change in top 25 country involvement over time

Figure 5 displays the development of extracted country of origin over time. Most documents that supply information of at least one country in the <contrib> tag are of US American origin. Although China has been a relatively small contributor till 1999, content distribution rapidly increased between 2000-2009. Today, China represents the second most frequently detected country of origin.

Besides the total number of countries supplying content to PMC the network structure of affiliations and countries has increased steadily. Today’s scientific community is heavily connected. Besides being the most prevalent publishers, US-American affiliations most often appear in papers by international collaborations. Most frequent country cooperations were found for the USA and the United Kingdom, the USA and China, as well as the USA and Germany.

### Special character and character representation

A widely used PMC standard to code special characters is the hexadecimal system (e.g.: ’α’ for ’$\alpha$’). Another common character coding system is the Unicode system (e.g.: ’\u03b1;’ for ’$\alpha$’). Both coding systems contain representations of characters from nearly any alphabet, mathematical characters and symbols, as well as text formatting characters.

Two technically clean approaches exist to display mathematical content in nearly any browser. The Mathematical Markup Language (Ausbrooks et al. 2014) and the MathJax Tex library (The MathJax Consortium 2018) are widely used and compatible methods. MathML is made to enable mathematics to be served, received, and processed on the World Wide Web, just as HTML has enabled this functionality for text (Ausbrooks et al. 2014). MathJax is an open-source JavaScript display engine for LaTeX, MathML, and AsciiMath notation that works in all modern browsers (The MathJax Consortium 2018). Within PMC, MathML and MathJax formulas are used with hexadecimal coded special letters and LaTeX annotation.

*JATSdecoder* transforms MathML and MathJax formulas into a plain text representation and unifies many pre-detected synonyms of character representations (bold, cursive and other equivalents of the same character) to simple Unicode letter representations.

**Fig. 6:**
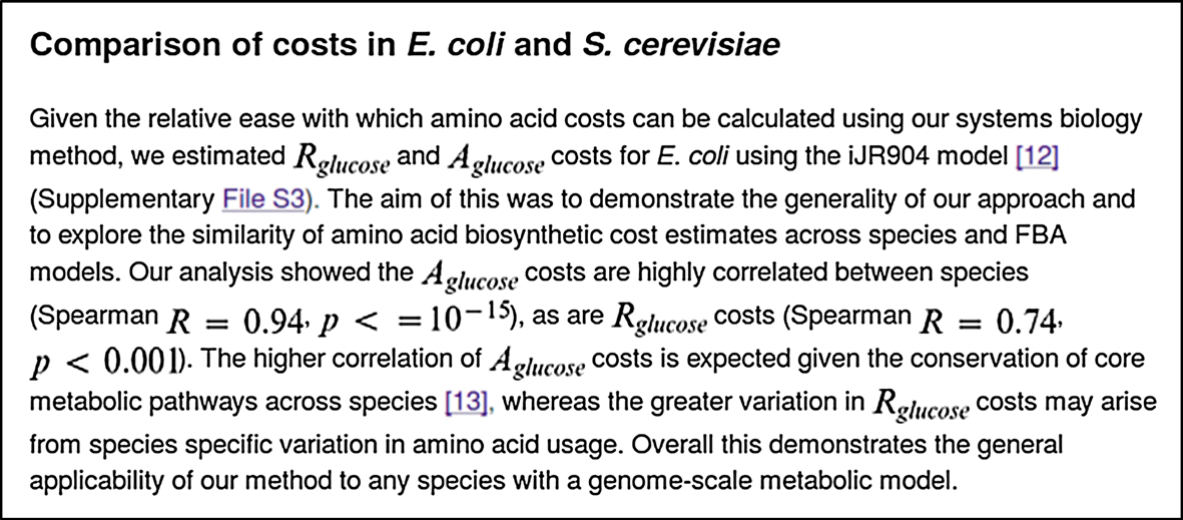
Example of <inline-formula> use with hyper referenced pixeled pictures from Barton et al. (2010)

Some documents contain an unfavorable approach to store special characters, mathematical content like formulas and/or statistical results within text. A hyper-referenced picture is included with an <inline-formula> tag. When using a browser, this representation becomes obvious by zooming into the page. An example is given in Fig. 6. The bold pixelated text parts are represented as pictures that do not resize into line height when increasing the view size (source: Barton et al. 2010. Evolutionary systems biology of amino acid biosynthetic cost in yeast. PloS One, 5(8)).

## Discussion

The PubMedCentral database is a rich and valuable resource for contextual text mining approaches on scientific content. A huge amount of scientific articles and materials from different areas of research is easily accessible in a rather consistent, pre-structured form. Although explicitly not designed to aid text mining, the NISO-JATS system enables a valid extraction of metadata and text parts with *JATSdecoder*. *JATSdecoder* copes with a wide range of possible implementations of NISO-JATS and facilitates customizable mirroring of scientific publication processes. It supports text analytical tasks on full documents or specific text parts or metadata only. Still, all results reported here rely on the assumption, that every way of tagging the targeted content was taken into consideration and correctly handled.

Although most tags are used quite consistently nowadays, any text analytical research on PMC’s content has to face several challenges and limitations, depending on the topic of interest. Some tags are not being used consistently by all distributors, some change over time (e.g. retracted documents), some are set incorrectly, probably due to human error.

The full PMC text corpus is fluid and increasingly up to date, but also provides historic documents that are being made available by many distributors. Some documents received a post-processing when their status changed (retraction/correction). The document collection is highly selective for the biomedical and health sciences, as it only contains content with an open access license. It also contains some documents from other research areas (e.g. physics). Further, most of the detected journals only provide very few documents. About half of all journals that provide content to the database only link to 10 or fewer files. An analysis including the content of these journals should always consider this fact.

Most of the journals that publish with a high frequency and their editors obviously have a Western origin. The frequent absence of an author identification number is still a crucial element for analyses on publications by a specific author or network. Before the U.S. National Institutes of Health (NIH) and its National Library of Medicine (NLM) launched the modern PubMed system, the math, physics and computer science community already solved this problem with the creation of ’arXiv’ in the early 1990s (Harrison and Harrison 2016). Harrison and Harrison ’s claim: *”The time has come for ’DOIs for authors”* ought to be implemented for authors and editors as soon as possible. The probability that more than one author bears the same name inside the scientific community or even the same subdiscipline grows every day. A retrospective discrimination of author and editor identity will become an increasingly difficult task.

Neither keyword nor subject tag supply uniform information about the topic relatedness of a document. Researchers working with article subsets should consider various tags as in- and exclusion criteria for their selection. The selection of articles covering a certain topic should be performed by a combined search task on title, abstract, keywords and/or the subject tag.

The implementation of a general topic extracting algorithm, that accumulates information from title, subject keyword and/or abstract and text, to select content, would highly facilitate selection. It could be of future interest, if ’topic models’ performed on <subject> and <kwd> tag lead to comparable results if generated from title, abstract and/or full text. Such algorithms would enable an easy visualization of the evolution of certain research topics over time.

The presentation of statistical results as images is a struggling task for automated text analysis on numerical content and should be avoided by distributors. None of the research using text analysis on scientific online literature has considered this issue. There are several open source optical character recognition (OCR) software packages available (e.g. tesseract), which may serve for a pixel to text conversion for pixeled content. The precision of such conversion could be content of future research, but should never be expected to be perfect. Simple reports on statistical parameters or results (e.g.: $R=.74$ or *p*<0.001) should be quite well extractable with OCR. Their ability to recognize complex formulas will be limited until trained adequately.

The steady increase of the amount of research findings published every year complicates the discrimination of valid and robust results from weak and questionable evidence. Text mining offers some great opportunities for selection processes and may boost a self-correcting culture in science.

## Data Availability

*JATSdecoder* software is freely available at: https://github.com/ingmarboeschen/JATSdecoder Skripts to reproduce this and other analyses performed with JATSdecoder are stored at: https://github.com/ingmarboeschen/JATSdecoderEvaluation.
